# Impact of improved minimally invasive anterior vitrectomy on the prognosis of patients with malignant glaucoma

**DOI:** 10.1186/s12886-024-03310-2

**Published:** 2024-01-24

**Authors:** Xuequn Liu, Yan Hu, Tian Yang, Zhong Wang, Zhen Wang

**Affiliations:** 1 Aier Eye Hospital of Nanchang, 330002 Nangchang, China; 2 Nanyang Eye Hospital of Henan Province, 473000 Nanyang, China

**Keywords:** Malignant glaucoma, Improved minimally invasive anterior vitrectomy, Phacoemulsification, Intraocular lens implantation, Retrospective observational study

## Abstract

**Background:**

The importance of communicating the anterior chamber and vitreous cavity for managing malignant glaucoma (MG) is widely recognized. This study investigated the impact of improved minimally invasive anterior vitrectomy (IAV) on the prognosis of MG.

**Methods:**

This retrospective interventional study included patients with MG who underwent conventional surgery or improved minimally IAV in Nanchang Aier Eye Hospital between January 2011 and April 2021. For the improved step, a small amount of triamcinolone acetonide was injected into the vicinity of the iris. Then, the residual vitreous body adhering to triamcinolone acetonide was excised. Comparisons were made using repeated measures ANOVA, t-test, and chi-squared test.

**Results:**

Thirty-one eyes from 26 patients were included: 15 eyes from 13 patients in the conventional group and 16 eyes from 13 patients in the IAV group. The 1-week, 1-month, and 3-month intraocular pressure (IOP) and the 3-month mean central anterior chamber depth were comparable between the two groups (all *P* > 0.05). The conventional group showed one eye with intraoperative vitreous hemorrhage and two eyes with postoperative re-shallowing of the anterior chamber; such events did not occur in the IAV group, and none developed corneal endothelial decompensation, IOL deviation, suprachoroidal hemorrhage, or retinal detachment during treatment and follow-up.

**Conclusion:**

Patients with MG who undergo improved minimally IAV might have similar postoperative IOP and central anterior chamber depth compared with conventional surgery but with reduced complications such as intraoperative vitreous hemorrhage and postoperative re-shallowing of the anterior chamber. Improved minimally IAV might be an alternative surgery for MG.

**Supplementary Information:**

The online version contains supplementary material available at 10.1186/s12886-024-03310-2.

## Backgrounds

Malignant glaucoma (MG), also known as ciliary block glaucoma or aqueous misdirection syndrome, is a form of rare secondary angle-closure glaucoma that causes serious vision damage [1–3]. MG is generally considered a complication after inner eye surgery such as trabeculectomy (Trab), phacoemulsification (Phaco), peripheral iridectomy, and retinal vitreous surgery [1–3], but MG can also be observed after laser iridotomy, laser posterior capsulotomy, laser scleral flap suture lysis, laser cyclophotocoagulation, and topical use of miotics [4–6]. It may even form spontaneously [7, 8]. The incidence of MG after glaucoma surgery is about 2.16-2.30% [9, 10]. 

The treatment of MG includes medication, laser, and surgery, ranging from vitreous humor aspiration and anterior vitrectomy to Phaco combined with anterior vitrectomy. Since MG is rare, it is difficult to acquire a large sample size and perform multicenter clinical trials. Several authors studied the pathogenesis, disease characteristics, and treatment of MG, but reaching a consensus on the surgical timing and technique for treating MG remains challenging. Nevertheless, since 2000, many authors have emphasized the importance of communicating the anterior chamber and vitreous cavity for the management of MG [6, 11], including vitrectomy-Phaco-vitrectomy [12], pars plana anterior vitrectomy, hyaloido-zonulectomy, and iridectomy [13], and modified partial pars plana vitrectomy combined with Phaco [14]. 

At the authors’ hospital, the method described by Sharma et al. [12] has been used since 2010 for the three-step surgical management of MG, achieving good surgical results. Nonetheless, problems such as intraoperative vitreous hemorrhage and postoperative anterior chamber-vitreous cavity obstruction have been observed. Therefore, since the end of 2017, Sharma’s three-step approach has been improved to reduce intraoperative and postoperative complications while ensuring the success rate of surgical anatomy.

Therefore, this study aimed to investigate the prognosis of improved minimally invasive anterior vitrectomy (IAV) for treating MG.

## Methods

### Study design and population

This retrospective interventional study included patients with MG admitted to the Department of Glaucoma of Nanchang Aier Eye Hospital between January 2011 and April 2021. Patients treated before the end of 2017 underwent the conventional three-step surgery, and those treated after 2017 underwent the improved minimally IAV surgery.

The inclusion criteria were (1) meeting the diagnostic criteria of MG [uniform shallow anterior chamber or disappearance of the anterior chamber after intraocular surgery, including Trab, glaucoma drainage device implantation, and Phaco (with or without goniosynechialysis, GSL)], (2) elevated or normal intraocular pressure, and (3) medical treatment cannot control the disease or medication can control the disease but without being taken continuously or the patient has a drug allergy. The exclusion criteria were (1) pupillary block, (2) suprachoroidal hemorrhage, (3) choroidal effusion, (4) lens subluxation, or (5) follow-up < 3 months [15]. 

This study complied with the Declaration of Helsinki and was approved by the ethics committee of Nanchang Aier Eye Hospital. The requirement for informed consent was waived by the ethics committee due to the retrospective nature of the study.

### Data collection definition

The collected data included age, sex, type of glaucoma, phakic eyes, eyes with intraocular lens (IOL) implantation, type of surgery, postoperative intraocular pressure (IOP) at 1 week, 1 month, and 3 months postoperative, best-corrected visual acuity (BCVA) before and after surgery, anterior chamber depth at preoperative and 3 months postoperative, and complications. The types of glaucoma included acute angle-closure glaucoma and chronic angle-closure glaucoma. The types of surgery included Trab, Phaco combined with IOL implantation (Phaco + IOL), Trab combined with Phaco and IOL (Phaco + IOL + Trab), Phaco and IOL combined with GSL (Phaco + IOL + GSL), laser peripheral iridotomy (LPI).

### Intervention

All patients underwent anterior vitrectomy or anterior vitrectomy combined with phacoemulsification and intraocular lens (IOL) implantation. Retrobulbar anesthesia was used for most patients (peribulbar anesthesia was used for some patients with advanced glaucoma) using 2–3 ml of a 1:1 mixture of 2% lidocaine and 0.75% bupivacaine. A 25 G (or 23 G) puncture cannula was used to make a puncture port in the pars plana of the ciliary body below the temporal (4.0 mm posterior to the corneal and scleral margin and 3.5 mm for an IOL), and the cannula was indwelled. A part of the central vitreous body was removed using a vitrector to reduce the volume of the vitreous body, thereby lowering IOP and forming an anterior chamber.

Phacoemulsification was completed, and the IOL was implanted (omitted for eyes already with an IOL). A peripheral iridectomy was performed using a vitrector if there was no iris peripheral incision.

The anterior chamber was perfused through the lateral corneal incision, and the vitrector was used to remove the peripheral basal vitreous body, lens suspensory ligament, and part of the equatorial capsule under the iris peripheral incision through the puncture cannula in the pars plana of the ciliary body, thereby forming a channel between the anterior chamber and the vitreous cavity.

The improved step: a small amount of triamcinolone acetonide suspension was injected into the vicinity of the iris peripheral incision through the anterior chamber, with the triamcinolone acetonide particles being seen to enter the vitreous cavity through the iris peripheral incision. Then, the residual vitreous body adhering to the triamcinolone acetonide particles near the iris peripheral incision was further excised.

All patients were operated on by the same experienced operator using the Alcon’s Centurion phacoemulsification 23 G vitrectomy system or Accurus 25 G vitrectomy system, with a cutting rate of 3000–4000 times/min and negative pressure of 50–250 mmHg. All patients were given tobramycin, and dexamethasone eye drops for 4 weeks after surgery, gradually reducing doses. Non-steroidal medication was used for topical eye instillation for 4 weeks. Systemic corticosteroids were used for 3–5 days without postoperative cycloplegic agents.

### Outcomes

The outcomes included visual acuity (no light perception, light perception; hand motion; counting fingers to 0.04, 0.05–0.25, and 0.3 and above), intraocular pressure (IOP), anterior chamber depth, at 1 week after discharge and 1 and 3 months after surgery.

### Statistical analysis

SPSS 24.0 (IBM Corp., Armonk, N.Y., USA) was used for data analysis. The continuous variables were presented as mean ± standard deviation (SD) and analyzed using the repeated measures ANOVA and the t-test. Categorical data were expressed as n (%) and compared by the chi-squared test. Two-sided P-values < 0.05 were considered statistically significant.

## Results

There were 13 patients (15 eyes) in the conventional surgery group (two males and 11 females; 60.9 ± 11.2 years of age) and 13 patients (16 eyes) in the IAV group (three males and 10 females; 46.8 ± 21.2 years of age). The basic characteristics between the two groups of patients were similar (all *P* > 0.05), except for the status of the lens (*P* = 0.015) (Table 1).

**Table 1:** The basic characteristics between the two groups of patients

Variables	Conventional group	IAV group	P value
	*n* = 13, eyes = 15	*n* = 13, eyes = 16	
Age	60.9 ± 11.2	46.8 ± 21.2	0.044
Sex			1.000
Female	11 (81.8%)	10 (76.9%)	
Male	2 (18.2%)	3 (23.1%)	
Type of angle-closure glaucoma			0.484
Acute	0	2 (12.5%)	
Chronic	15 (100%)	14 (87.5%)	
Status of the lens			0.015
Phakic	8 (53.3%)	15 (93.7%)	
Pseudophakic	7 (46.7%)	1 (6.3%)	
Type of surgery	(13.3%)		0.275
Trab	11 (73.3%)	14 (87.5%)	
Phaco + IOL	1 (6.7%)	0	
Phaco + IOL + GSL	1 (6.7%)	0	
Phaco + IOL + Trab	0	1 (6.3%)	
LPI	0	1 (6.3%)	

Trab, trabeculectomy; Phaco, phacoemulsification; IOL, intraocular lens; GSL, goniosynechialysis; LPI, laser peripheral iridotomy

**Fig. 1:**
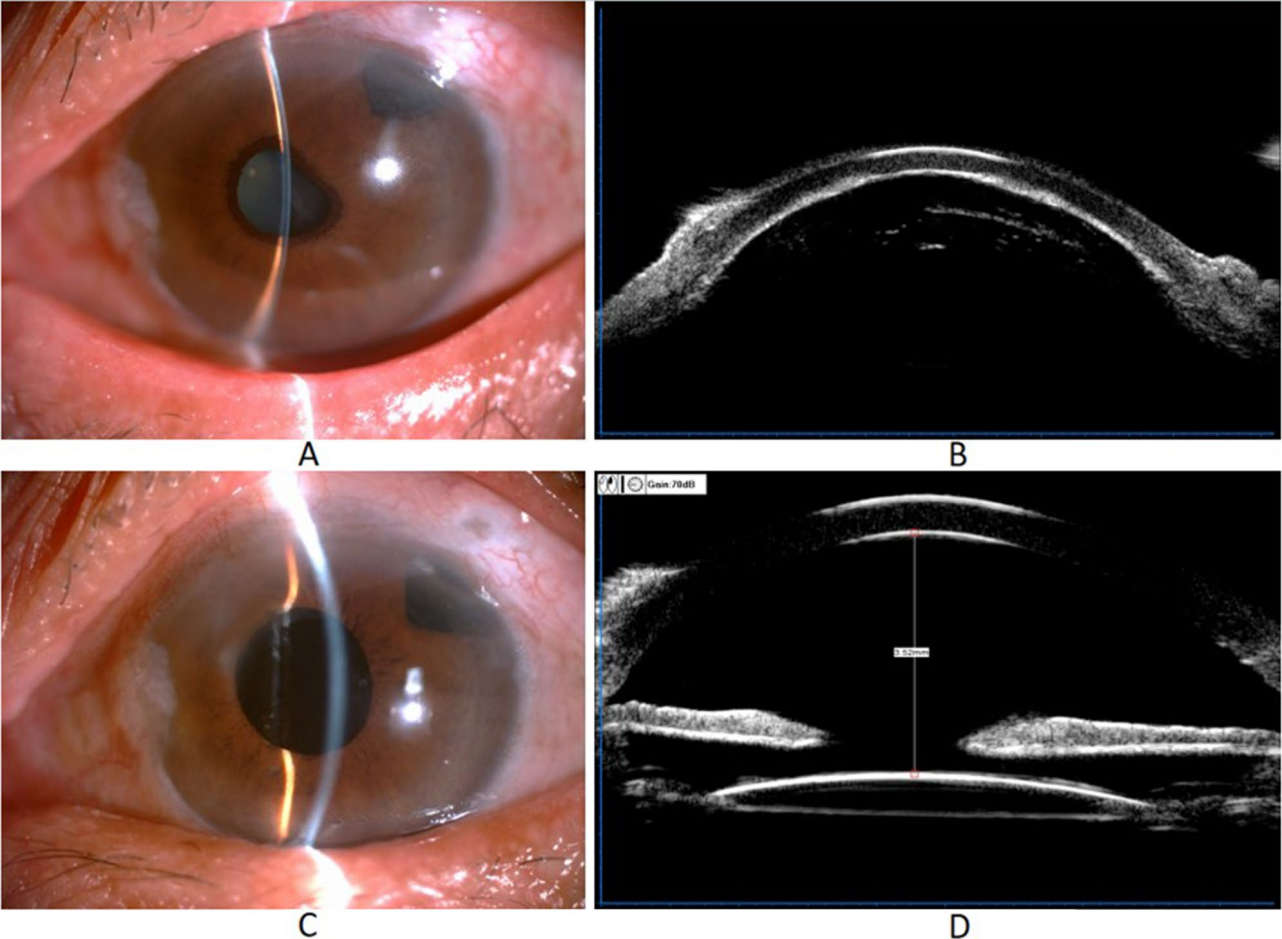
Slit-lamp examination and ultrasound biomicroscopy(UBM)before (**A**-**B**) and after (**C**-**D**) improved minimally invasive anterior vitrectomy in a patient with malignant glaucoma

The visual acuity of patients in the two groups before [LogMRA: 1.10 (0.90, 1.40) vs. 2.00 (0.65, 2.30), *P* = 0.599] and after surgery [LogMRA: 0.70 (0.20, 0.90) vs. 0.90 (0.43, 2.23) *P* = 0.078] were comparable. Moreover, compared with preoperative, the IOP at 1 week, 1 month, and 3 months postoperative was significantly decreased in both groups (all *P* < 0.05), while comparable between groups (all *P* > 0.05). One patient had to undergo repeat Trab due to IOP being out of control 2 weeks after surgery. The 3-month postoperative mean central anterior chamber depth was significantly increased in both groups (all *P* < 0.05) but comparable between groups (all *P* > 0.05) (Table 2). Figure 1 presents the slit-lamp examination and anterior chamber depth of a 64 years female from the IAV group.

**Table 2:** Comparison of patients’ visual acuity, intraocular pressure, and anterior chamber depth before and after surgery

Outcomes	Conventional group	IAV group	P_group_ value
	*n* = 13, eyes = 15	*n* = 13, eyes = 16	
Visual acuity, logMAR＊			
Preoperative	1.10 (0.90, 1.40)	2.00 (0.65, 2.30)	0.599
Postoperative	0.70 (0.20, 0.90)	0.90 (0.43, 2.23)	0.078
No light perception			-
Preoperative	0	1	
Postoperative	0	1	
Light perception			-
Preoperative	0	1	
Postoperative	0	0	
Hand motions			-
Preoperative	2	5	
Postoperative	1	5	
Counting fingers to 0.04			
Preoperative	3	2	
Postoperative	2	3	-
0.05–0.25			
Preoperative	9	4	
Postoperative	6	4	-
0.3 and above			
Preoperative	1	3	
Postoperative	6	5	
IOP (mmHg)			
Preoperative	43.9 ± 14.2	29.1 ± 15.3	0.009
1-week postoperative	15.6 ± 3.8^a^	16.7 ± 5.6^a^	0.535
1-month postoperative	16.0 ± 3.2^a^	17.1 ± 3.5^a^	0.359
3-month postoperative	16.2 ± 2.2^a^	17.1 ± 2.6^a^	0.294
Central anterior chamber depth (mm)			
Preoperative	0.67 ± 0.61	0.65 ± 0.58	0.908
3-month postoperative	3.32 ± 0.20^b^	3.31 ± 0.35^b^	0.917

*Note* ＊VA was evaluated in all patients using the Snellen’s original test with conversions to decimal and logMAR scales for statistical analyses. Lower VAs were calculated as follows: counting fingers, 1/100 (logMAR 2); hand motions, 1/200 (logMAR 2.3); light perception, 1/666 (logMAR 2.8), and no light perception, 0 (logMAR 3)

^a^*P* < 0.05 vs. preoperative; ^b^*P* < 0.001 vs. preoperative. IOP, intraocular pressure. P _group_, the significance of the difference between groups

In the conventional group, one eye had an intraoperative vitreous hemorrhage, and two eyes had anterior chamber re-shallowed 1–2 weeks after surgery (whose anterior chamber deepened after yttrium-aluminum-garnet (YAG) laser incision of the organized membrane at the iris peripheral incision). By contrast, such events did not occur in the IAV group. For other early complications, such as corneal edema (severe corneal edema in five eyes occurred in MG with the disappearance of the anterior chamber), the cornea became transparent after 3–10 days. During the treatment and follow-ups, none of the patients experienced corneal endothelial decompensation, IOL deviation, suprachoroidal hemorrhage, or retinal detachment.

## Discussion

In this study, patients with MG who underwent improved minimally IAV might have similar postoperative IOP and central anterior chamber depth compared with conventional surgery but reduced complications such as intraoperative vitreous hemorrhage and postoperative re-shallowing of the anterior chamber. These findings might support the improved minimally IAV as an alternative surgery for MG.

MG was first described by Von Graefe in 1869, but its exact pathogenesis has not been fully elucidated. It is believed to be related to anatomical abnormalities of the lens-ciliary body-anterior vitreous body, particularly the anterior rotation of the ciliary process that leads to aqueous misdirection and retention in the vitreous cavity or vitreous hydration and swelling, thereby causing the lens-iris septum to move forward and the anterior chamber to become shallow [16, 17]. Quigley et al. [18] argued that choroidal dilation leads to vitreous compression and lens-iris septum advancement. Therefore, some authors suggested replacing the term “MG” with “chronic aqueous misdirection syndrome” to define its pathology and pathogenesis more accurately [5]. 

The clinical treatment of MG includes medication, laser, and surgery. Medication includes cycloplegic agents, drugs that inhibit aqueous humor production, hypertonic agents, and corticosteroids. Laser therapy includes laser posterior capsule and anterior vitreous limiting membrane incision and transscleral diode laser cyclophotocoagulation [19]. Surgical intervention is necessary when a patient responds poorly to drugs or laser therapy (with various durations of observation). All patients in this study were treated with medication. Among the 31 eyes, 26 responded to the drugs (the anterior chamber deepened and IOP decreased), and five experienced the disappearance of the anterior chamber, which was believed to require emergency surgery.

There are numerous surgical options for treating MG. In 1964, Chandler [20] reported vitreous water-bag aspiration that can immediately relieve the lens-iris septum advancement caused by vitreous humor retention. It is simple to perform, causes small trauma, and does not need vitrectomy equipment. On the other hand, it has a low success rate, and MG is likely to recur (three eyes underwent this surgery in the present study and had a recurrence). Thomas et al. [21] reported that an elderly MG patient with IOL and iris peripheral incision, who was refractory to medical therapy and could not tolerate surgery, recovered the anterior chamber by needle puncture in the pars plana under a slit-lamp.

According to Lois et al. [22] and Zarnowski et al. [23] MG in eyes with an IOL can be treated by performing suspensory ligament-anterior vitreous limiting membrane-anterior vitrectomy through a limbal incision, anterior chamber, and iris peripheral incision. Sharma et al. [12] introduced a three-step surgical approach for treating MG in phakic eyes, namely, central vitrectomy, Phaco, IOL implantation, and anterior vitreous-anterior vitreous limiting membrane-suspensory ligamentectomy. Debrouwere et al. [24] proposed that complete vitrectomy combined with peripheral iridectomy-suspensory ligamentectomy (combined with Phaco for phakic eyes) is the most effective treatment of MG. The surgical method of the IAV group in this study was modified based on Sharma’s three-step approach. The surgical results were consistent with the conclusions of other studies, suggesting that the key to surgery’s success lies in building an anterior chamber-vitreous cavity channel that lasts long.

Schmidt et al. [25] reported that after complete vitrectomy and anterior vitreous limiting membrane-suspensory ligament-peripheral iridectomy, one eye out of five underwent a second anterior vitreous limiting membrane-suspensory ligament-peripheral iridectomy. Dave et al. [26] reported four cases of recurrence after vitreous body-anterior vitreous limiting membrane-suspensory ligament-peripheral iridectomy due to inflammatory exudative membrane coverage (*n* = 3) and IOL loop block (*n* = 1). In this study, two eyes in the conventional group had slight shallowing of the anterior chamber (IOP unchanged) within 2 weeks after surgery. Suspected exudative membranes were observed at the lens capsule plane under the iris peripheral incision (possibly due to small anterior and posterior traffic junctions and postoperative inflammation), blocking the aqueous humor from flowing to the anterior chamber. As a result, the aqueous humor was retained again in the vitreous cavity, causing the IOL-iris septum to move forward and, therefore, the anterior chamber to become shallow again. In the IAV group, we took advantage of the anti-inflammatory effect of triamcinolone acetonide and the “marking” effect of the triamcinolone acetonide particles on the anterior vitreous body to verify the patency of the intraoperatively built anterior and posterior channels and to reduce surgical inflammation.

Some studies reported the intraoperative complications of vitreous hemorrhage [14, 15]. One eye in the conventional group also developed vitreous hemorrhage, which could be related to the congestion and edema of the ciliary process after the MG attack. In addition, the vitrector had a low cutting rate and high negative pressure, possibly resulting in accidental injury to the ciliary process, causing hemorrhage. In the IAV group, vitrectors with a high cutting rate and low negative pressure were used to avoid such accidents.

Consistent with some retrospective studies [27–29] and a case report [30] on surgical timing, patients with MG should be operated on as soon as possible (particularly for those with primary angle-closure glaucoma), even if some patients respond to medical treatment. There are several reasons. First, early surgical intervention can reduce the loss of corneal endothelial cells; long-term attachment or even adhesion of the iris and the anterior lens capsule to the corneal endothelium will seriously damage the corneal endothelial cells. Second, the surgery’s difficulty can be reduced by intervening early when the cornea is relatively clear for operation. Third, early recovery of the anterior chamber can mitigate postoperative inflammatory response, facilitating the formation and maintenance of filtering blebs. Fourth, the long-term use of atropine can cause allergy and uncertainty of adherence, which might result in repeated attacks of MG. Fifth, with the dual guarantee of accurate biometrics and proficient surgical skills, modern cataract surgery can produce good postoperative visual quality.

The major change compared with the traditional technique is the use of triamcinolone acetonide, which provides a visual and definite marker for building an anterior chamber-vitreous cavity channel. All residual vitreous bodies adhering to the triamcinolone acetonide particles near the iris peripheral incision could be further removed, ensuring a more thorough surgery. In addition, the anti-inflammatory effect of triamcinolone acetonide reduces the postoperative inflammatory response in the eye and contributes to the formation and maintenance of filtering blebs, which reduces uncontrolled postoperative intraocular pressure. Besides the use of triamcinolone acetonide, the vitrectomy technology with high frequency and low negative pressure enhances the accuracy of removing the lens suspensory ligament and basal vitreous body under the iris peripheral incision, avoiding damage to the ciliary process and retina (which reduces the occurrence of intraocular hemorrhage and retinal detachment) and ensuring the stability of the IOL. Still, the two procedures had similar effectiveness, but more complications were observed in the conventional group compared with the IAV group. Fewer complications are a possible consequence of using IAV, but the number of events in the conventional group and the sample size were small. Therefore, additional studies are necessary for confirmation.

The limitations of this study lie in its small sample size, sample selection bias, and bias arising from the operator’s learning curve. There was a difference in patient age between the two groups, but that difference could not be controlled for because of the small number of patients. More patients will be included in the future to confirm the surgical outcomes. Moreover, we hope that this improvement can provide more and better options for the surgical treatment of MG.

## Conclusion

In conclusion, patients with MG who underwent improved minimally IAV might have similar postoperative IOP and central anterior chamber depth with conventional surgery but reduced complications such as intraoperative vitreous hemorrhage and postoperative re-shallowing of the anterior chamber. Nevertheless, these findings need further confirmation by a prospective, multicenter study with a large sample size.

### Electronic supplementary material

Below is the link to the electronic supplementary material.

Supplementary Material 1

## Data Availability

All data generated or analyzed during this study are included in this published article and supplementary information files.

## Abbreviations

IAV: invasive anterior vitrectomy
MG: malignant glaucoma
Trab: trabeculectomy
Phaco: phacoemulsification
IOL: intraocular lens
BCVA: best-corrected visual acuity
LPI: laser peripheral iridotomy
IOP: intraocular pressure
SD: standard deviation
YAG: yttrium-aluminum-garnet
