# Supplementary material for: Impact of improved minimally invasive anterior vitrectomy on the prognosis of patients with malignant glaucoma
Source: BMC Ophthalmol. 2024 Jan 24;24:39. doi: 10.1186/s12886-024-03310-2 (PMC10809461; doi:10.1186/s12886-024-03310-2)
Supplement: Supplementary file 1 — Supplementary Material 1 [file 12886_2024_3310_MOESM1_ESM.docx]

**Supplementary materials**

**Surgical method**

All patients underwent anterior vitrectomy or anterior vitrectomy combined with phacoemulsification and intraocular lens (IOL) implantation. Retrobulbar anesthesia was used for most patients (peribulbar anesthesia was used for some patients with advanced glaucoma) using 2-3 ml of a 1:1 mixture of 2% lidocaine and 0.75% bupivacaine. A 25 G (or 23 G) puncture cannula was used to make a puncture port in the pars plana of the ciliary body below the temporal (4.0 mm posterior to the corneal and scleral margin, and 3.5 mm for an IOL) and the cannula was indwelled. A part of the central vitreous body was removed using a vitrector to reduce the volume of the vitreous body, thereby lowering intraocular pressure and forming an anterior chamber.

Phacoemulsification was completed, and the IOL was implanted (omitted for eyes already with an IOL). A peripheral iridectomy was performed using a vitrector if there was no iris peripheral incision.

The anterior chamber was perfused through the lateral corneal incision, and the vitrector was used to remove the peripheral basal vitreous body, lens suspensory ligament, and part of the equatorial capsule under the iris peripheral incision through the puncture cannula in the pars plana of the ciliary body, thereby forming a channel between the anterior chamber and the vitreous cavity.

The improved step: a small amount of triamcinolone acetonide suspension was injected into the vicinity of the iris peripheral incision through the anterior chamber, with the triamcinolone acetonide particles being seen to enter the vitreous cavity through the iris peripheral incision. Then, the residual vitreous body adhering to the triamcinolone acetonide particles near the iris peripheral incision was further excised.

All patients were operated on by the same experienced operator using the Alcon’s Centurion phacoemulsification 23 G vitrectomy system or Accurus 25 G vitrectomy system, with a cutting rate of 3000-4000 times/min and negative pressure of 50-250 mmHg. All patients were given tobramycin and dexamethasone eye drops for 4 weeks after surgery, gradually reducing doses. Non-steroidal medication was used for topical eye instillation for 4 weeks. Systemic corticosteroids were used for 3-5 days without postoperative cycloplegic agents.
